# Genome-wide pharmacogenetics of anti-drug antibody response to bococizumab highlights key residues in HLA DRB1 and DQB1

**DOI:** 10.1038/s41598-022-07997-5

**Published:** 2022-03-11

**Authors:** Daniel I. Chasman, Craig L. Hyde, Franco Giulianini, Rebecca D. Danning, Ellen Q. Wang, Timothy Hickling, Paul M Ridker, A. Katrina Loomis

**Affiliations:** 1 Division of Preventive Medicine, Brigham and Women’s Hospital, Boston, MA USA; 2 Harvard Medical School, Boston, MA USA; 3 Pfizer Inc., 1 Portland Street, Cambridge, MA USA; 4 Pfizer Inc., New York, NY USA

**Keywords:** Drug development, Pharmacogenetics, Pharmacogenomics, Dyslipidaemias

## Abstract

In this largest to-date genetic analysis of anti-drug antibody (ADA) response to a therapeutic monoclonal antibody (MAb), genome-wide association was performed for five measures of ADA status among 8844 individuals randomized to bococizumab, which targets PCSK9 for LDL-C lowering and cardiovascular protection. Index associations prioritized specific amino acid substitutions at the *DRB1* and *DQB1* MHC class II genes rather than canonical haplotypes. Two clusters of missense variants at *DRB1* were associated with general ADA measures (residues 9, 11, 13; and 96, 112, 120, 180) and a third cluster of missense variants in *DQB1* was associated with ADA measures including neutralizing antibody (NAb) titers (residues 66, 67, 71, 74, 75). The structural disposition of the missense substitutions implicates peptide antigen binding and CD4 effector function, mechanisms that are potentially generalizable to other therapeutic mAbs.

**Clinicaltrials.gov**: NCT01968954, NCT01968967, NCT01968980, NCT01975376, NCT01975389, NCT02100514.

## Introduction

Injectable monoclonal antibodies (mAbs) are an increasingly prioritized option for highly specific and safe therapeutics. Yet, the efficacy of mAb therapeutics may be attenuated by anti-drug antibodies (ADAs) that may accelerate mAb clearance and/or interfere with binding to the target, the latter termed neutralizing anti-drug antibodies (NAbs)^1–6^. Understanding genetic influences on ADA response can help address molecular mechanisms underlying this immunogenicity with potential implications for improving development of mAb therapeutics and their clinical translation.

To date, genetic analyses of ADA response to mAb therapeutics have been limited to relatively small samples, typically using candidate gene approaches, although more recent studies have taken a genome-wide association (i.e. GWAS) perspective. The genetics of ADA response with the α-IL17A mAb secukinumab^7^ as well as with the α-TNFα mAbs infliximab and adalimumab have highlighted haplotypes in the HLA class II *DRB1* and *DQB1* genes and also a haplotype of *DRA1*^8–10^. Outside of the major histocompatibility complex (MHC), the V158F variant of the IgG Fc fragment receptor (*FCGR3A*), which has a highly plausible mechanistic link to ADA response, has been associated with both ADA and treatment response to therapeutic MAbs^11–14^.

Bococizumab is an injectable, humanized mAb targeting proprotein convertase subtilisin–kexin type 9 (PCSK9) for lowering of LDL cholesterol (LDL-C) in the prevention of cardiovascular disease (CVD)^15^. In SPIRE, a family of double-blind, randomized, placebo controlled phase 3 trials, bococizumab was overwhelmingly effective at lowering LDL-C and also showed evidence of reducing incident CVD risk. However, high-titer ADAs were elicited among some trial participants and, among those with the ADA maximum titer in the top 10% after 1 year of therapy, NAbs to PCSK9 were also induced with concomitant attenuation of LDL-C lowering^16^. Bococizumab development was discontinued due to this unanticipated loss of efficacy, alongside a higher incidence of ADA and a higher rate of injection-site reaction compared with other agents in this drug class.

Here, we explore the genetic determinants of ADAs and NAbs for bococizumab among individuals in SPIRE with European ancestry, the majority subset. In this much larger sample for pharmacogenetics of mAb therapies than has been available previously, genome-wide significant associations primarily implicated multiple variants in the MHC region, concentrated around the HLA *DRB1* and *DQB1* genes. The associations reveal critical roles for specific clusters of missense variation in the immune response whose impact may be interpreted in the context of structural models of DRB1 and DQB1 proteins.

## Methods

### Study population

Genetic analysis was performed among participants in the SPIRE family of randomized, double-blind, placebo-controlled trials examining the efficacy of bococizumab for various outcomes. One sub-family of SPIRE, SPIRE lipid-lowering, examined LDL-C reduction with bococizumab for 1 year of follow-up or less as indicated for various entry criteria and was completed as planned^16^. Among the SPIRE lipid-lowering trials, SPIRE-HR targeted individuals with high cardiovascular risk, in spite of intensive statin treatment; SPIRE-LDL targeted individuals with LDL-C ≥ 70 mg/dL and multiple cardiovascular risk factors; SPIRE-FH targeted individuals with familial hypercholesterolemia; and SPIRE-LL targeted individuals for whom lipid lowering beyond benefit with statin was indicated. The second sub-family of SPIRE, SPIRE CVO, examined reduction in cardiovascular outcomes for two levels (SPIRE1 and SPIRE2) of cardiovascular risk and was terminated after a median follow up of 7 to 12 months of the planned 3.9–4.8 year follow-up due to discontinuation of drug development^17^. Overall, approximately 74.8% (range 71.4–87.4% by sub-study) of SPIRE participants randomly allocated to bococizumab provided consent and a blood sample for exploratory genetic analysis. All SPIRE participants included in this analysis provided informed written consent for genetic analysis. All data collection and analysis was conducted in accordance with relevant guidelines and regulations for secondary use of deidentified samples, and was approved by the Institutional Review Board of Brigham and Women’s Hospital.

### Laboratory measures

Antidrug antibodies (ADA) were determined by a validated quasi-quantitative assay as described at baseline and at regular intervals during follow-up in both the SPIRE lipid-lowering and SPIRE CVO trials^16, 17^. ADA titers are reported as the log_2_ of the experimental measure, and ADA positive status was determined as ADA titers (as log_2_) ≥ 6.23 (= 75 exponentiated) at any time during the study for individuals without pre-existing ADA titers or > 1.58 unit increase in titer from baseline if subject was ADA-positive at baseline. In the SPIRE lipid-lowering trials, ADA-positive samples were further tested for neutralizing antibodies (NAb) using a validated assay as described, also reported after log_2_ transformation^16^. NAb titers among individuals with negative ADA status were set to zero. Individuals with NAb titers (as log_2_) ≥ 1.58 (= 3 exponentiated) over follow up were assigned to NAb positive status. LDL-C levels at baseline and throughout follow up were determined by direct assay from blood draw after at least 10 h fasting.

### Genotype data

Participant DNA was extracted by Brooks Life Sciences, Indianapolis, IN. Genotyping was performed at Illumina using the Omni Express-Exome-10 K version 1.4 chip. Genotypes were called with Illumina GenomeStudio (Illumina, San Diego) software. Quality control procedures were applied to the raw genotype data using PLINK 1.9^18^. Samples were retained if genotypes were available for > 98% of the markers, while markers were retained if genotypes were available for > 90% of the retained samples. Samples were excluded with high heterozygosity (based on chi-square test of squared z-score of the inbreeding coefficient F) or mismatch of sex inferred from the genotypes with the clinical record. Multidimensional scaling (MDS) was applied among the combination of the remaining samples and the 1000G multi-ancestry reference panel to 3982 single nucleotide polymorphisms (SNPs) selected with high Fst (> 0.17) in the 1000G reference panel^19^. Among the first 3 components of the MDS decomposition, individuals were provisionally considered to have European ancestry if they mapped within 6 standard deviations of an ellipsoid around the centroid of individuals from the 1000G with known European ancestry. Of these, individuals with self-reported European ancestry were retained. Finally, among pairs of related individuals with inferred IBD > 0.185, only the individual with fewer missing genotypes was retained. In this sample, markers were excluded with p value < 10^–6^ for test of Hardy–Weinberg equilibrium. Overall, from 13,283 samples with DNA, 13,198 (99.4%) had high-quality genotyping and unambiguous sex, among whom 9808 (73.8%) were designated with European ancestry and unrelated, and finally 8844 had been randomized to active bococizumab treatment (which was the preferentially genotyped arm in several of the trials). Within this sub-sample, 965,074 genotyped markers were available.

Additional genotype data in the sub-sample with European ancestry were derived by imputation. Across the entire genome, genotypes were imputed to the Haplotype Reference Consortium (HRC) panel through initial phasing by Eagle (v.2.3) and imputation with minimac (v.3), as implemented on the University of Michigan imputation server^20^. Additional genetic variation was imputed in the Major Histocompatibility Complex (MHC) region with SNP2HLA using the T1DGC reference panel that is expected to yield high accuracy imputation^21^. Some alleles from the MHC region imputation are coded in terms of amino acid substitutions or as canonical HLA haplotypes. Some of these alleles represent the presence of one or more amino acids compared with their absence. Genetic analysis of the MHC region exclusively used data imputed with SNP2HLA.

### Statistical analysis

In order to fully capture genetic influences on effects of bococizumab on the immune system, five response variables were considered: ADA positive or NAb positive during follow-up (1 = positive, 0 = negative); ADA maximum titer or NAb maximum titer (continuous) were the maximum titers respectively among individuals designated as ADA or NAb positive—both were rank normal transformed for genetic analysis; ADA maximum titer in the top 10% indicated whether or not (1/0) the ADA maximum titer over follow-up was in the top decile of the ADA maximum titers among individuals with positive ADA status. The latter was included because individuals who were NAb positive were enriched among those with ADA maximum titer in the top 10% in the SPIRE lipid-lowering trials^16^ and could serve as a proxy of NAb in the larger sample from the SPIRE1 and SPIRE2 sub-studies, in which NAb status was not available for most subjects. Additional analyses examined the absolute and/or fractional change in LDL-C from baseline to 12 months follow-up, with rank normal transformation.

Genetic association testing assumed an additive relationship between the number of inherited alleles and mean or log-odds outcome in either linear or logistic regression frameworks as appropriate, unless otherwise noted. The clinical covariates age, sex, smoking status, and triglycerides at baseline were included in these regressions when they were significantly associated with the outcome. Similarly, analysis that included samples from multiple SPIRE sub-studies included a sub-study indicator variable. In addition, principal components of European population sub-stratification computed with EIGENSTRAT were included as covariates^22^. Genome-wide association with experimental genotypes or imputed maximum likelihood allele dose was performed with PLINK v. 1.9 or ProbABEL v 0.5.0, respectively^23^. Similarly, association testing of imputed maximum likelihood MHC alleles was performed with PLINK 1.9.

Using the same regression formalism as for the primary analysis, successive rounds of conditional analysis were performed to identify secondary signals within the MHC region. For each round, index SNP(s) from the previous round(s) were included as covariates for regression analysis of all other MHC variants. The most significant SNP at each round was retained and the process continued until the lead conditional SNP no longer met nominal significance after accounting for multiple testing across all MHC variants examined using a permutation procedure, i.e. until corrected p value = p_corr_ > 0.05, as follows. Over 1000 iterations, phenotype values were re-assigned without replacement at random to individuals, the primary regression was performed to identify a most significant primary index SNP, followed by successive rounds of conditional analysis, including the most significant index SNPs from previous rounds as covariates. These regressions provided the null distribution for the analytic p values of lead SNPs at each conditional round. The analytic p values of the conditional associations from the unpermuted data for each phenotype were compared to these null distributions to derive empirically adjusted p values.

Structural annotations were made with PyMol (v.2.0.3)^24^ using publicly available models 6CQN for DRB1^25^, 6DIG for DQB1^26^, and 3S4S for the complex of DRB1 and CD4^27^ from the Research Collaboratory for Structural Bioinformatics Protein Data Bank (RCSB PDB)^28^. Additional programming was performed in R^29^ including the Manhattan plots that were made with the R package CMPlot^30^.

## Results

### Demographics and outcomes

Demographic features of all SPIRE participants as well as participants for each sub-study with available genetic data and verified European ancestry are shown in Table S1. SPIRE participants randomly allocated to treatment had mean age 63.3 years (range of mean across sub-studies 57.2–64.0 years), were 28.6% female (23.4–39.3%), with mean BMI 30.6 (28.6–32.0) kg/m^2^, and prevalence of smoking 22.7% (15.0–25.0%). After up to approximately 12 months of follow-up (“Methods”), 32.4% of available SPIRE participants randomly allocated to bococizumab met the criteria for a positive ADA response, varying across sub-studies from 25.9 to 52% (Table S2). Among 1048 available participants on treatment with a positive ADA response, 312 (29.8%) from SPIRE-HR, SPIRE-LDL, SPIRE-FH, and SPIRE-LL tested positive for presence of neutralizing antibodies (NAbs), a binary outcome variable (0/1). Genetic analysis was performed with 5 related phenotypes, using the maximum available sample for each (“Methods”): (1) positive v. negative ADA status (N = 8774), (2) positive v. negative NAb status (N = 1048), (3) ADA maximum titer over follow-up among those with positive for ADA (N = 2841), (4) maximum NAb titer over follow-up among those with positive NAb status (N = 312), and (5) ADA maximum titer top 10% of those with positive ADA status (N = 2841) (Table S3). Of standard clinical variables, 4 (age, sex, smoking status, and triglycerides) were associated with one or more of the ADA related outcomes, accounting for multiple testing (p < 0.001, Table S4, “Methods”).

### Genome-wide association analysis

**Figure 1:**
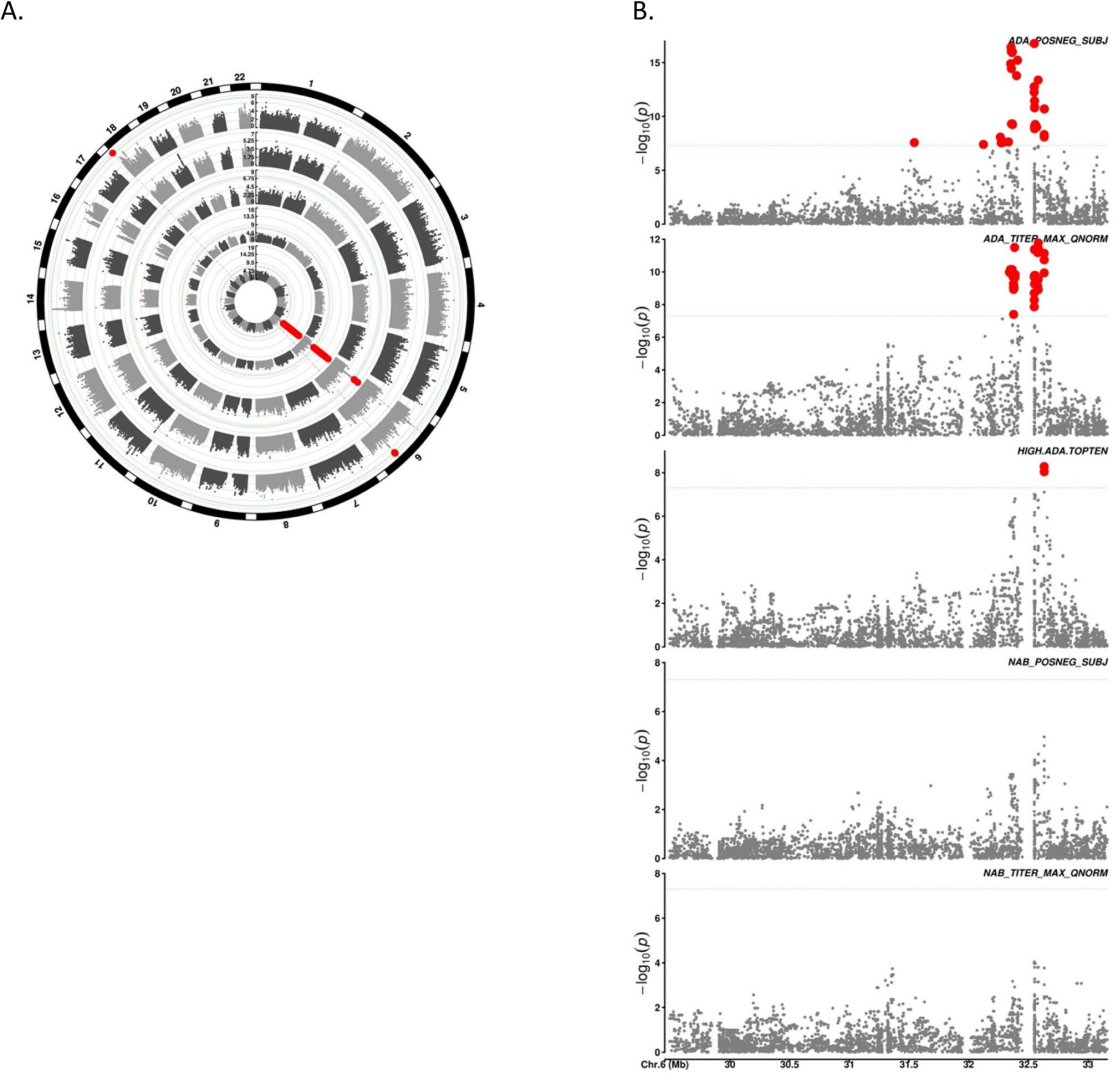
Genome-wide associations with anti-drug antibody response to bococizumab in SPIRE. (**A**) Circular Manhattan plot of genome-wide associations using genome-wide genotype data imputed to the HRC reference panel. Each ring corresponds to an anti-body response phenotype ordered from the central ring toward the perimeter as ADA positive, ADA maximum titer, ADA maximum titer top 10%, NAb positive, NAb maximum titer (see “Methods”). Thin green dashed lines indicate genome-wide significance threshold (p = 5 × 10^–8^) for each genome-wide scan. Red dots indicate SNPs reaching genome-wide significance. See Supplementary Figure S1 for corresponding quantile–quantile (QQ) plots. (**B**) Manhattan plot restricted to the MHC region (chr6:29-33 Mb) derived from MHC-specific imputation. Antibody response phenotypes as indicated are in the same order, top to bottom, as phenotypes in (**A**) center to perimeter. Thin green dashed line and red dots as in (**A**).

There were independent genome-wide significant (p < 5 × 10^–8^) associations (with minor allele frequency at least 5%) for all phenotypes except NAb positive status, and all but two of these associations mapped to the major histocompatibility complex (MHC) locus on chromosome 6 (“Methods”, Fig. 1). Quantile–quantile plots for all phenotypes were well-behaved with genomic control lambdas all near one (Figure S1). The index, i.e. the independent and most significant, associations for each phenotype are presented in Table 1. Both genome-wide significant loci outside the MHC were observed for maximum NAb titer, for which the sample size was only 312 and both had allele frequency only slightly greater than the 5% threshold. One maps to the *COLEC12* gene encoding a member of the C-lectin family on chromosome 18, while the other maps to an intergenic region of chromosome 6, remote from the MHC. Although sex was associated with ADA response in SPIRE, there were no associations on the X chromosome or interactions of SNP genotypes with sex reaching the genome-wide significance standard.

**Table 1:** Primary genome-wide significant associations for anti-drug antibody phenotypes.

Phenotype	Variant type	N or N case/N control	Variant ID	chr:pos (hg19)	Nearest gene(s)	Imputation^@^	A1/A2^	A1 freq	Beta (SE) S.D. or OR (95%CI)	p
ADA positive	AA variant^#^	2841/5933	AA_DRB1_120_32657518_N	6:32549539	*DRB1*	MHC	A/P*	0.13	0.66 (0.57–0.71)	1.68E−17
	AA variant^#^		AA_DQB1_75_32740612	6:32632633	*DQB1*	MHC	L/V	0.25	1.25 (1.16–1.34)	4.84E−09
	SNP		rs3093664	6:31544641	*TNF*	MHC	G/A	0.08	1.40 (1.25–1.58)	2.69E−08
ADA max titer	SNP	2841	rs7756741	6:32583197	*DQA1/DRB1*	MHC	A/T	0.33	0.19 (0.03)	1.68E−12
	SNP		rs3763313	6:32376470	*BTNL2*	MHC	A/C	0.25	0.17 (0.03)	3.98E−08
ADA max titer top 10%	AA variant^#^	286/2555	AA_DQB1_71_32740624_KD	6:32632633	*DQB1*	MHC	P/A*	0.25	1.72(1.43:2.06)	5.24E−09
NAb max titer	SNP	312	rs78928382	18:370039	*COLEC12*	HRC	G/A	0.06	−0.85 (0.15)	1.87e−08
	SNP		rs111663071	6:120683067	*intergenic*	HRC	A/G	0.06	0.65 (0.12)	2.35E−08

Index SNPs determined by clumping with LD R^2^ = 0.1. ^#^Notation is AA_gene_residue_nucleotide b.36_encoded amino acid(s)^21^^@^Imputation using the genome-wide HRC panel (HRC) or the MHC panel (MHC). ^A1 = coded (i.e. effect) allele/A2 = non-coded allele, indicated as alternative single letter amino acid code for AA variants or alternative nucleotide for SNPs. *AA in variant ID present (P) or absent (A). Covariates were age, sex, study, PCs 1–5.

The remainder of the index genome-wide significant associations were clustered in the MHC, spanning approximately chr6:29-33 Mb (build hg19), as revealed by specialized imputation targeting this region (“Methods”^21^). For ADA positive status, the most significant index association mapped to the *DRB1* gene encoding the presence or absence of asparagine at amino acid 120 (AA_DRB1_120_32657518_N; OR [95% CI] = 0.64[0.57:0.71]; p = 1.7 × 10^–17^). Additional index associations with lesser but still genome-wide significance were an amino acid variant, AA_DQB1_75_32740612, in the *DQB1* gene, and a non-coding SNP, rs3093664, nearest the *TNF* gene. The top index genome-wide associations with ADA titer max, rs7756741 and rs3763313, were non-coding SNPs nearest the *DQA1* and *BTNL2* genes, respectively. Finally, the sole genome-wide significant index variant for ADA maximum titer top 10% was another amino acid substitution in DQB1, AA_DQB1_71_32740624_KD. This same SNP was also the index association for NAb positive status, although not reaching genome-wide significance (p = 1.1 × 10^–5^).

**Figure 2:**
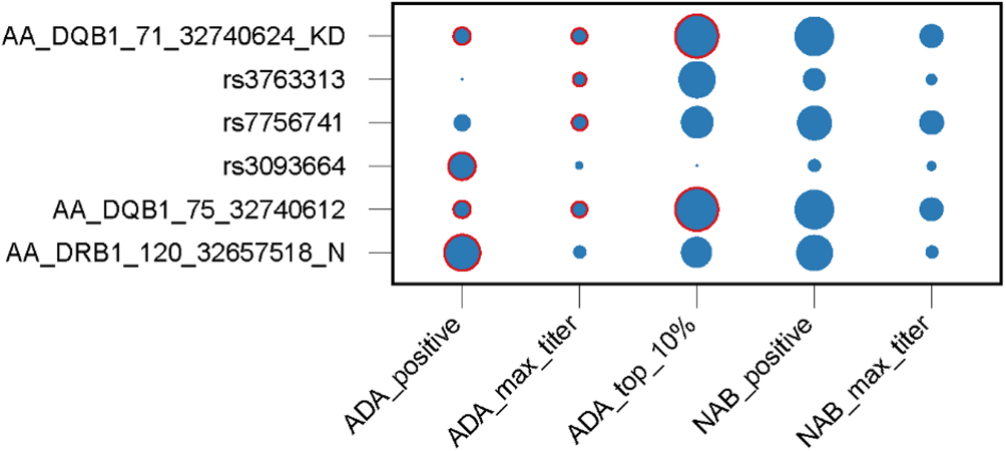
Effects of index SNPs across the several measures of immunogenicity. Diameters of circles are proportional to the magnitude of variant effects (i.e. beta coefficients). Circles with red outlines indicate associations reaching genome-wide significance. See also Table S5.

At the index variants, there were several patterns of association across the ADA and NAb phenotypes (Fig. 2, Table S5). However, essentially identical patterns for AA_DQB1_71_32740624_KD and AA_DQB1_75_32740612 reflect the essentially perfect LD between these variants, while partially related patterns for rs7756741 and AA_DQB1_75_32740612 reflect their partial LD (r^2^ = 0.60).

In spite of the association of sex with ADA response, there were no nominally significant interactions with sex at the index variants (Table S6). There was also no evidence for non-additive character in associations at the index SNPs after allowing for multiple testing (Table S7). The proportion of variance explained for the index SNPs in the MHC was 1.2% for ADA maximum titer and 1.7% (pseudo R^2^) for ADA positive status.

Some of the index variants were in near perfect LD (r^2^ > 0.9) with additional variants, highlighting the relevance of combinations of amino acid substitutions and haplotypes to each of the outcomes (Table 2). Thus, index non-synonymous variant AA_DRB1_120_32657518_N (p = 1.7 × 10^–17^) was in high LD with additional protein coding substitutions at DRB1 residues 96, 112, 166, and 180, as well as nonsense variation at multiple residues C-terminal to residue 96 (Table S8). AA_DRB1_120_32657518_N was also in high LD with canonical haplotype DRB1 0407, although the association of this allele with ADA positive status was less significant (p = 1.4 × 10^–13^) than for the amino acid substitution at residue 120. AA_DQB1_75_32740612, the second most significant association for ADA positive status, had proxies that were amino acid substitutions at *DQB1* not only at residue 71 (as above) but also at residues 66, 67, and 74. This LD cluster was identical to the cluster indexed on ADA maximum titer top 10%. Finally, the non-coding index variant for ADA maximum titer, rs7756741, was in strong LD with amino acid substitutions at DRB1 residues 9, 11, and 13.

**Table 2:** Proxies (LD r^2^ > 0.9) for MHC region index variants.

Phenotype	Index variant	Proxies	
		Residues^	Haplotypes
ADA positive	AA_DRB1_120_32657518_N	DRB1_96, DRB1_112, DRB1_166, DRB1_180	HLA_DRB1_0407
	AA_DQB1_75_32740612*	DQB1_66, DQB1_67, DQB1_71, DQB1_74	N/A
	rs3093664	N/A	N/A
ADA max titer	rs7756741	DRB1_9, DRB1_11, DRB1_13	N/A
	rs3763313	N/A	N/A
ADA max titer top 10%	AA_DQB1_71_32740624_KD*	DQB1_66, DQB1_67, DQB1_74, DQB1_75	N/A

*Same LD cluster. ^Residues affected by non-synonymous proxies.

### Secondary MHC signals from conditional analysis

Sequential rounds of conditional analysis revealed significant multivalent genetic influences on ADA response phenotypes after empirical correction for multiple testing across the entire ~ 4 Mb of the MHC region, i.e. p_corr_ < 0.05 (Table 3). For ADA positive status, SNPs in the MHC region remained significant through at least 10 rounds of conditioning. Similarly, for ADA maximum titer, SNPs remained significant after 5 rounds of conditioning. However, there were no significant secondary signals for ADA maximum titer top 10% nor NAb positive status after conditioning on the most significant SNP in the MHC region. There was minimal pairwise LD (expressed as r^2^) between the primary index SNPs and the secondary conditional SNPs, which spanned most of the MHC, as much as 2.9 Mb from the index SNPs (Table 3, Table S9). Including both primary and secondary signals in the MHC, the proportions of variance in the measures of immunogenicity increases to 3.2% for ADA maximum titer and 3.6% (pseudo R^2^) for ADA positive status (up from 1.2 and 1.7% of variance explained, respectively).

**Table 3:** Additional variants from sequential rounds of conditioning.

Phenotype (primary variant)	Round	Variant	hg19 chr 6 position	Distance (bp) from primary variant^	p	p_corr_*
**ADA positive (AA_DRB1_120_32657518_N)**						
	1	rs3093664	31544641	1004898	1.16E−06	< 0.001
	2	rs3130215	31525911	1023628	2.54E−06	< 0.001
	3	rs9268500	32376516	173023	5.46E−06	< 0.001
	4	rs9268543	32384800	164739	1.11E−08	< 0.001
	5	rs9469069	31866417	683122	4.64E−05	< 0.001
	6	rs3763313	32376470	173069	1.49E−04	0.001
	7	rs3130062	31525911	1023628	1.01E−03	0.006
	8	rs29220	29589665	2959874	1.08E−03	0.010
	9	rs2267635	29592430	2957109	5.86E−04	0.013
	10	rs4713420	30993566	1555973	2.40E−03	0.029
**ADA max titer (rs7756741)**						
	1	AA_DRB1_13_32660109_FG	32552130	31067	4.69E−05	0.002
	2	rs2523453	31368124	1215073	1.49E−05	0.002
	3	rs17496549	32409707	173490	4.92E−04	0.007
	4	AA_C_9_31347600_S	31239620	1343577	9.42E−04	0.015
	5	rs2240804	30920889	1662308	1.93E−03	0.041
	6	rs3763355	32786881	− 203684	2.48E−03	0.056
	7	rs2395171	32394536	188661	3.01E−03	0.093

*p value corrected for multiple testing from 1000 iterations of resampling (Methods). ^positive values are 5' direction, negative are 3'.

### Candidate gene analysis

Only a few genetic variants with previously reported ADA associations were available in the SPIRE dataset (Table S10). Of these, there was significantly reduced ADA with inheritance of DRB1*03 (OR [95%CI] 0.82 [0.69–0.97], p = 0.02) and equivalently, in this dataset, with DRB1 03:01, that had been implicated for infliximab^9^. There was no association of these alleles with NAb status. There was also no association at DQA1*05 that had been implicated for adalimumab and also infliximab^10^. The genetic variant implicated in previous genetic studies of ADAs for monoclonal antibody treatments and mapping to chromosome 1, *FCGR3A* rs396991 (V158F)^11–14^, is known to be challenging to genotype accurately, and was neither genotyped directly nor available in the reference panel for imputation^31, 32^.

### Genetics of longitudinal ADA titer

Data on the immune response to bococizumab were collected at prespecified intervals throughout the approximately 1 year of follow-up, but not at each visit for all participants. Nevertheless, there is a clear increase over time in the fraction of treated SPIRE participants that were judged ADA positive (Table S11A)^16^; and the genetic associations at the lead variants with ADA are already manifest early during follow-up. For example, the lead variant for ADA status (AA_DRB1_120_32657518_N_A) is significant for ADA status already at week 12, and the lead variant for ADA maximum titer top 10% and NAb positive status (AA_DQB1_71_32740624_KD_P) is significantly associated with ADA status already at week 4. Similarly, the fraction with NAb positive status already rises significantly at week 4 for some of the index SNPs. (Table S11B).

### Effects on LDL-C response to bococizumab

We investigated the extent to which genetic influences on ADA response also influenced the change in LDL-C levels with bococizumab treatment. Among 3220 individuals in SPIRE-CVO with non-missing information on change in LDL-C level, there were no genome-wide significant associations with either absolute or fractional change in LDL-C over 1 year of follow-up. Among the primary variants identified for the various ADA and/or NAb responses, none was significantly associated with LDL-C response (Table 4). Similarly, there were no significant effects on LDL-C response among variants identified in the conditional analysis (data not shown). It remained possible that there may be SNPs in the MHC that are nominally associated with LDL-C change but not identified in analysis of the various ADA responses. We identified (independent) variants in this region that had nominal association (i.e. p < 0.01) with either absolute or fractional LDL-C change in the SPIRE-CVO sub-group of 7725 participants with available LDL-C change information after a year of follow-up. These SNPs were then tested for association with ADA response in the SPIRE-CVO subgroup of 3220 individuals with available ADA response information. None of these candidates was significant with the ADA and/or NAb response phenotypes after accounting for multiple testing.

**Table 4:** Genetic association with LDL-C response at primary index variants.

Discovery phenotype	Index variant	Beta (SE), p value*	
		Absolute LDL-C change	Fractional LDL-C change
ADA positive	AA_DRB1_120_32657518_N	0.011 (0.03), 0.74	− 0.019 (0.033), 0.58
	AA_DQB1_75_32740612	0.047 (0.0271),0.08	0.04592(0.0276), 0.10
	rs3093664	0.064 (0.045), 0.15	0.060 (0.045), 0.19
ADA max titer	rs7756741	0.039 (0.026), 0.13	0.045 (0.026), 0.08
	rs3763313	0.036 (0.027), 0.18	0.043 (0.027), 0.11

*From linear regression including covariates age, sex, smoking, study, and eigenvectors of population substructure. Units of SD after rank normal transformation of phenotype.

### Structural models of top HLA associations

**Figure 3:**
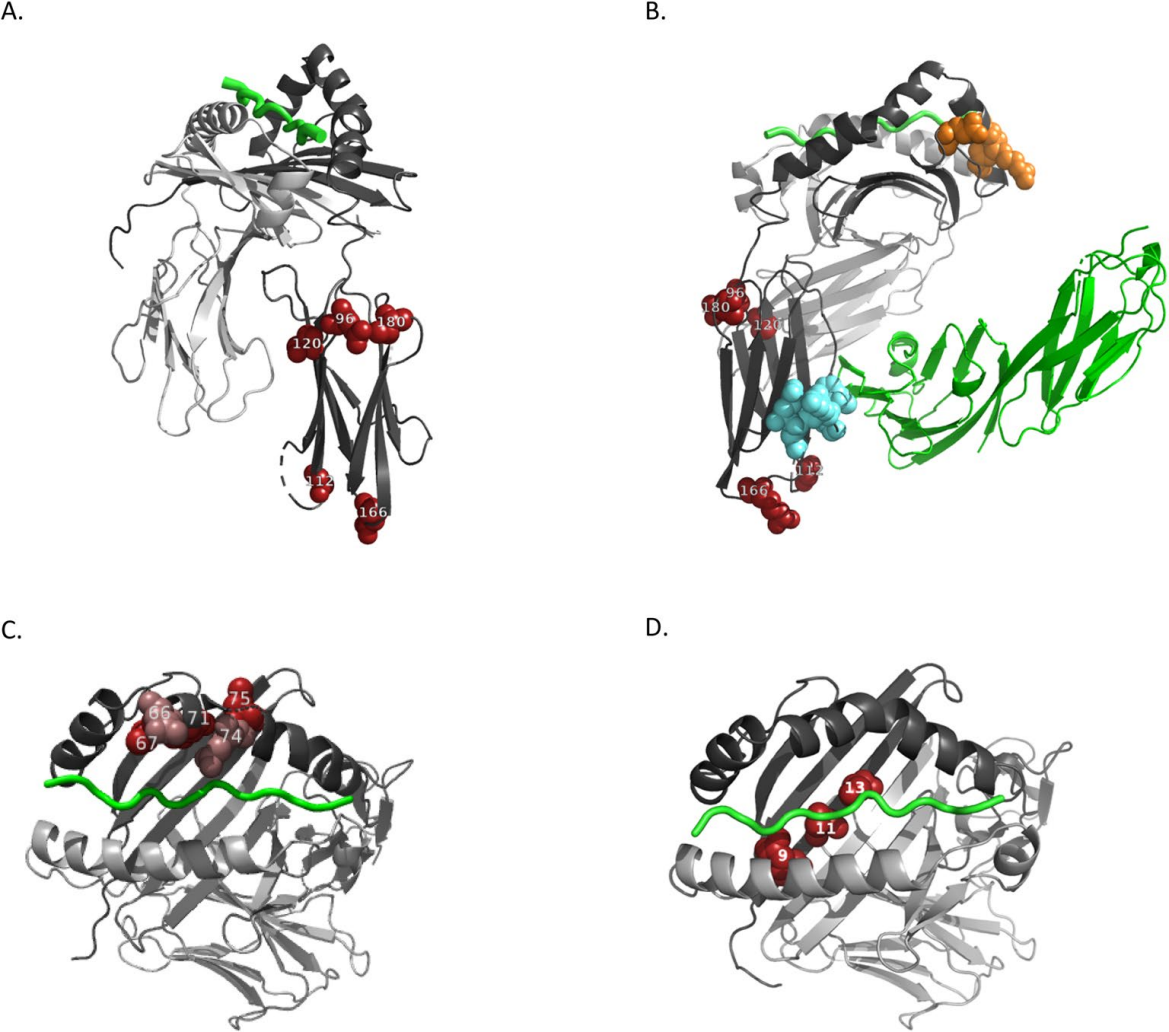
Mapping of index non-synonymous variants and their proxies to structural models of DR1 and DQ1. (**A**) ADA positive status on DR1. Index non-synonymous variant at DRB1 residue 120 and its proxies at residues 96, 112, 166, and 180 shown as red spheres are mapped onto the crystal structure of DR1 (PDB ID: 3S4S)^27^. DRA1 and DRB1 peptides are represented in light and dark gray ribbons, respectively. A 13 residue peptide in the peptide-binding grove is indicated by a green coil. The index variant and its proxies map to the β2 beta-sandwich domain of DRB1. (**B**) ADA positive status on DR1 with CD4. Same representation of the DRB1 index variant 96 and its proxies as in (**A**), but now including CD4, making contact with the β2 beta-sandwich domain of DRB1. Also shown are residues in DRB1 inferred from mutagenesis to interfere with CD4-mediated T-cell response from Brogdon et al. (residues, 46, 54, 55, 56, orange spheres)^34^ and Konig et al. (residues, 137–143, cyan spheres)^33^. (**C**) ADA maximum titer top 10% and NAb positive status on DQ1. Index non-synonymous variant at DQB1 residue 71 and its proxies at residues 66, 67, 74, and 75 are mapped onto the crystal structure of DQ1 (PDB ID: 6DIG)^26^, and represented as spheres alternating in darker and lighter red to aid visualization. DRA1 and DRB1 peptides are represented in light and dark gray ribbons, respectively. A 13 residue peptide in the peptide-binding grove is indicated by a green coil. (**D**) ADA maximum titer on DR1. Index non-synonymous variant at residue 13 and its proxies at residues 9 and 11 are mapped onto the crystal structure of DR1 (PDB ID: 6CQN)^25^, and represented as red spheres. DRA1 and DRB1 peptides are represented in light and dark gray ribbons, respectively. A 13 residue peptide in the peptide-binding grove is indicated by a green coil.

The structural context of the missense substitutions that comprise the top associations for the ADA phenotypes suggest likely molecular mechanisms involving CD4 interactions and peptide binding function of DRB1 and DQB1. The top index variant for ADA positive status, AA_DRB1_120_32657518_N, and its LD missense tags at residues 96, 112, 166, and 180, map to loops at the edges of the β2 beta sandwich domain of DRB1 (Fig. 3A). These residues are adjacent to each other, but each on a different strand. The solvent accessible face of the beta sandwich is thought to contact CD4 on the basis of structural analysis and mutagenesis studies^27, 33^ (Fig. 3B). The index variant for ADA maximum titer top 10%, AA_DQB1_71_32740624_KD (which is also the top but sub-genome-wide significant SNP for NAb positive status and in high LD with the second index for ADA positive status) and its missense LD proxies at residues 66, 67, 74, and 75 map to the proximal side of the β1 alpha helix that defines one rim of the DQB1 peptide binding groove (Fig. 3C). The index non-synonymous SNP for ADA maximum titer, which encodes variation in DRB1 at residue 13, and its LD proxies at residues 9 and 11 map to a single strand of the beta sheet that lines the base of the DRB1 peptide binding groove (Fig. 3D).

## Discussion

In this largest ever reported genome-wide association study (GWAS) of the immunogenicity of a therapeutic monoclonal antibody, there were genome-wide significant primary associations with four measures of ADA in the SPIRE trials of bococizumab: overall ADA status, ADA maximum titer among those with ADA positive status, ADA maximum titer in the top 10%, and NAb maximum titer among those with NAb positive status. The primary associations mapped to amino acid substitutions in the HLA DRB1 and DQB1 proteins rather than classical haplotypes of these loci and associated variation differed according to ADA measure. In conditional analysis, multiple secondary associations were also observed across the MHC region. These associations, together with the two non-coding genome-wide significant associations (rs3093664, rs3763313) that were not in LD with non-synonymous HLA variation, emphasize the multivalent nature and diversity of mechanism constituting the bococizumab ADA response. For example, the different genetic effects on measures of NAb compared with overall ADA imply, at a minimum, two classes of ADAs. The preeminence of common amino acid substitutions at key residues rather than canonical haplotypes^8–10^ suggests that immune reactivity to bococizumab is likely not a consequence of strong selective events such as infectious disease, at least in populations with recent European ancestry^35^.

Cumulatively, however, the genetic effects on the variance of ADA were minimal, in the range of 3–4%. The downstream consequences of these associations did not contribute substantially to the reduction in LDL-C lowering after 12 months. Perhaps unintuitively, this result is consistent. In the SPIRE trials, only participants on therapy whose ADA titers were in the top 10% of ADA response experienced a substantial reduction in therapeutic value, as reflected in reduction of both bococizumab concentration and LDL-C lowering after 1 year^16^. These effects, pertaining to individuals at the extremes of ADA concentration, were not strongly correlated with overall ADA titers.

Nevertheless, the pharmacogenetic associations may inform biological mechanisms underlying immune response to bococizumab and possibly other mAb therapeutics in general. In the structural models, DRB1 residues 11 and 13, for which non-synonymous substitutions are in high LD, are shared by pockets P4-P7 at the base of the binding groove where they form solvent inaccessible contacts with antigen peptide^36, 37^. Similarly, DQB1 residue 71 and linked substitutions at residues 66, 67, 74, and 75 form part of same set of pockets at the edge of the binding groove, also in direct contact with bound antigen peptide. Substitutions at both groups are critical in predicting peptide binding specificity from sequence and imply mechanisms related to antigen selection, presentation, and recognition by helper T-cells. Such mechanisms are consistent with observations in both humanized and fully human therapeutic mAbs that complementarity determining regions (CDRs), segments that are the most foreign, are also the most immunogenic and constitute T-cell epitopes^3–5, 38, 39^. Accordingly, NAbs for bococizumab, which interfere directly with PCSK9 binding, were observed among individuals with the highest titers of ADA^16^. However, it remains unclear whether the T-cell epitopes in bococizumab would derive from the murine sequences that persist in spite of humanization procedures, e.g. murine originating CDRs, or would also be relevant had the foreign sequences, e.g. CDRs, been derived from a full human mAb^3–5, 16, 38–40^.

The ADA response to bococizumab may also be related to intrinsic reactivity of the individual immune systems. Certainly, associations at DRB1 residue 120 and its proxies suggest a mechanism involving general CD4 function, which is critical to the activation of CD4 + helper T-cells, rather than specific T-cell epitopes. The structural models suggest that substitutions at residue 120 and its LD correlates may induce structural perturbations that are propagated through the β2 domain, modulating CD4 contacts and therefore CD4 + helper T-cell response. Further, there is striking similarity between the findings here and the prioritization of some of the same critical residues, i.e. rather than haplotypes, in *DRB1* and *DQB1* in susceptibility to autoimmune disorders^41–43^. In rheumatoid arthritis (RA), for example, although the RA susceptibility allele with the largest effect, val-his-lys-ala at DRB1 residues 11-13-71-74, is consistent with DRB1*0401, the combination val-(his/phe)-arg-ala is also very strongly associated and consistent with multiple DRB1 haplotypes, e.g. *0404, *0405, *0408, and *1001^43^. In type 1 diabetes, combinations of amino acids at only three residues, DQB1 residue 57, DRB1 13, and DRB1 71, account for most of the HLA-related risk even though these amino-acid based alleles do not correspond to specific DQB1 and DRB1 haplotypes^41^. DRB1 residue 13 is similarly prioritized over classical haplotype alleles in explaining systemic lupus erythematosus in an East Asian study population^42^.

While the strengths of this investigation are the genome-wide approach in the context of a randomized, placebo-controlled trial, and large sample size for ADA measures, there were also limitations. The main limitation is the relatively small sample size for analysis of the NAb response. NAb titers were essentially available only in the SPIRE lipid-lowering sub-sample due to early termination of the SPIRE-CVO. The lack of these measures in a large sample, and accompanying limiting power, may have precluded detection of secondary genetic associations with NAb status, as were found for ADA status. A second limitation is the restriction of the sample to individuals of European ancestry, which, for example, only captures a proportion of diversity at the HLA class II loci. SPIRE recruited participants with non-European ancestry, but the numbers available were not adequate for well-powered genome-wide genetic analysis.

Thus, the analysis of genetic influences on immune responses to bococizumab in SPIRE highlights susceptibilities to ADA and NAb induction focused on the *DRB1* and *DQB1* loci, both implicated in ADA response to other mAb therapeutics^7–9^. The patterns of association reveal a critical role for amino acid substitutions with structural dispositions related to formation of T-cell epitopes and CD4 binding of the encoded DRB1 and DQB1 proteins. Toward the goal of improving the development of mAb therapeutics, the findings may focus future research on mechanisms by which the highlighted residues enhance susceptibility to ADA reactions.

## Supplementary Information

Supplementary Information 1.

Supplementary Information 2.

## Data Availability

Upon request, and subject to review, Pfizer will provide the data that support the findings of this study. Subject to certain criteria, conditions, and exceptions, Pfizer may also provide access to the related individual anonymized participant data. See https://www.pfizer.com/science/clinical-trials/trial-data-and-results for more information.
