# Supplementary material for: Genome-wide pharmacogenetics of anti-drug antibody response to bococizumab highlights key residues in HLA DRB1 and DQB1
Source: Sci Rep. 2022 Mar 11;12:4266. doi: 10.1038/s41598-022-07997-5 (PMC8917227; doi:10.1038/s41598-022-07997-5)

SUPPLEMENTARY FIGURES FOR:

Genome-wide pharmacogenetics of anti-drug antibody response to bococizumab highlights key residues in HLA DRB1 and DQB1

Daniel I. Chasman[1], Craig L. Hyde[2], Franco Giulianini[1], Rebecca D. Danning[1], Ellen Q. Wang[3], Timothy Hickling[2*], Paul M Ridker[1], A. Katrina Loomis[2]

[1]Division of Preventive Medicine, Brigham and Women's Hospital, Boston MA; [2]Pfizer Inc., 1 Portland Street, Cambridge MA; [3]Pfizer Inc., New York, NY.

*During the design and implementation of the study

Key words

Bococizumab, pharmacogenetics, anti-drug antibody, neutralizing antibody, MHC, immunogenicity

*To whom correspondence should be addressed: dchasman@research.bwh.harvard.edu

Supplementary Figures

Supplementary Figure 1. Quantile-quantile (QQ) plots of -$\log_{10}$(p-value) from genome-wide scans for genetic association with ADA and NAb phenotypes as indicated. Plots restricted to variants with minor allele frequency > 5% and imputation quality R2 > 0.5.

A. ADA positive status (λ_{GC} = 1.01)

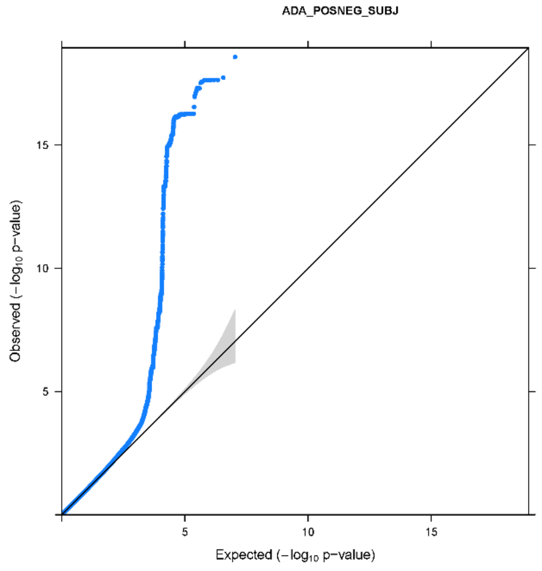

B. ADA maximum titer (λ_{GC}=1.01)

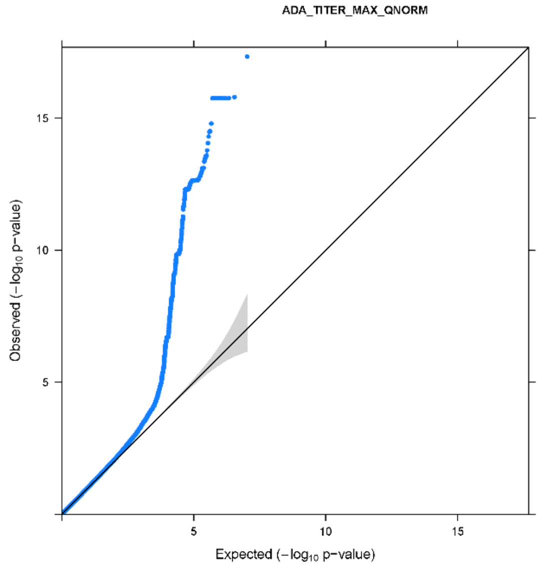

C. ADA maximum titer top 10% (λ_{GC}=1.01)

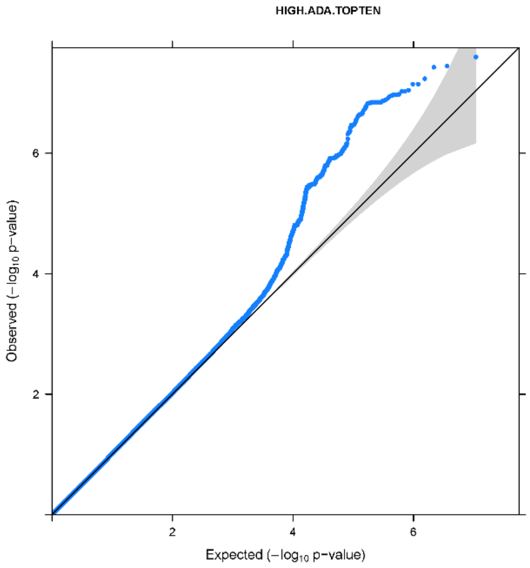

D. NAb positive status (λ_{GC}=1.01)

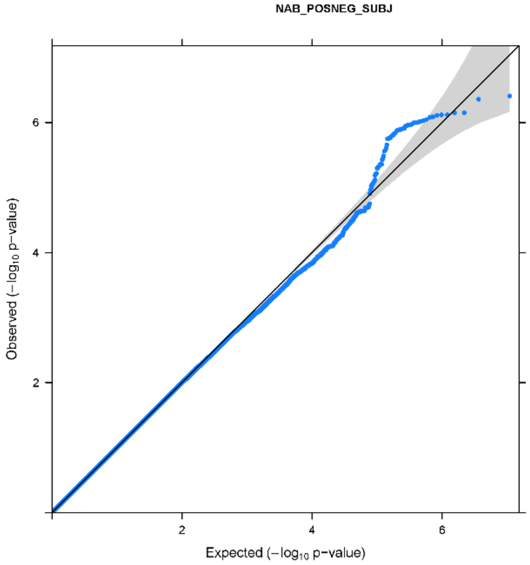

E. NAb maximum titer (λ_{GC}=1.06)

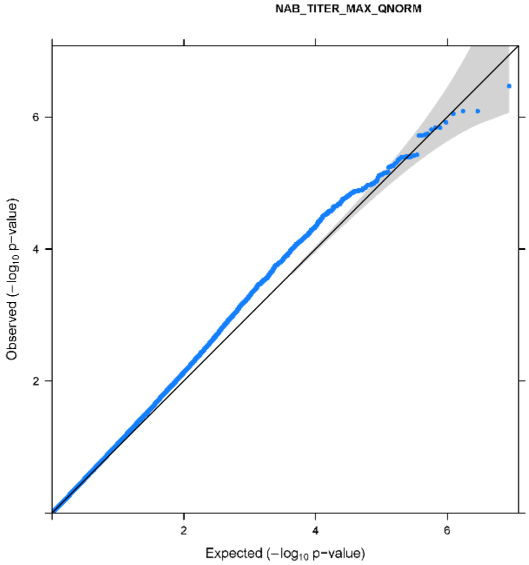

Supplement: Supplementary file 1 — Supplementary Information 1. [file 41598_2022_7997_MOESM1_ESM.pdf]
