# Supplementary material for: Genome-wide pharmacogenetics of anti-drug antibody response to bococizumab highlights key residues in HLA DRB1 and DQB1
Source: Sci Rep. 2022 Mar 11;12:4266. doi: 10.1038/s41598-022-07997-5 (PMC8917227; doi:10.1038/s41598-022-07997-5)
Supplement: Supplementary file 2 — Supplementary Information 2. [file 41598_2022_7997_MOESM2_ESM.pdf]

SUPPLEMENTARY TABLES FOR:

Genome-wide pharmacogenetics of anti-drug antibody response to bococizumab highlights key residues in HLA DRB1 and DQB1

Daniel I. Chasman[1], Craig L. Hyde[2], Franco Giulianini[1], Rebecca D. Danning[1], Ellen Q. Wang[3], Timothy Hickling[2*], Paul M Ridker[1], A. Katrina Loomis[2]

[1]Division of Preventive Medicine, Brigham and Women's Hospital, Boston MA; [2]Pfizer Inc., 1 Portland Street, Cambridge MA; [3]Pfizer Inc., New York, NY.

*During the design and implementation of the study

Key words

Bococizumab, pharmacogenetics, anti-drug antibody, neutralizing antibody, MHC, immunogenicity

*To whom correspondence should be addressed: dchasman@research.bwh.harvard.edu

Table S1. Demographic characteristics of overall sample available for genetic analysis and its sub-samples

Clinical variable	all studies	SPIRE sub-sample							
		SPIRE1	SPIRE2	SPIREHR	SPIRELDL	SPIREFH	SPIRELL	SPIRE CVO	SPIRE lipid-lowering
N	8844	4576	3203	173	532	102	258	7779	1065
baseline age (years)*	63.31 (9.31)	63.99 (9)	62.63 (9.58)	63.31 (9.27)	63.02 (8.81)	57.27 (11.24)	62.43 (10.03)	63.43 (9.27)	62.38 (9.58)
sex [N (%) female]	2529 (28.6%)	1074(23.47%)	1067(33.31%)	68(39.31%)	184(34.59%)	40(39.22%)	96(37.21%)	2141(27.52%)	388(36.43%)
BMI (kg/m2)*	30.56 (5.52)	30.4 (5.47)	30.5 (5.43)	30.69 (5.32)	32.04 (6.22)	28.63 (4.92)	31.53 (5.67)	30.44 (5.45)	31.37 (5.91)
smoking [N (%)]	2008(22.7%)	1027(22.44%)	802(25.04%)	26(15.03%)	84(15.79%)	16(15.69%)	53(20.54%)	1829(23.51%)	179(16.81%)
diabetes [N (%)]	3860(43.65%)	1994(43.58%)	1360(42.46%)	74(42.77%)	302(56.77%)	15(14.71%)	115(44.57%)	3354(43.12%)	506(47.51%)
LDL-C (mg/dl)*	111.24 (39.07)	93.1 (24.2)	135.34 (43.63)	107.21 (27.63)	106.39 (30.38)	141.61 (36.1)	134.35 (37.48)	110.49 (39.5)	116.68 (35.36)
HDL-C (mg/dl)*	48.37 (12.87)	48.53 (12.97)	48.17 (12.81)	48.71 (14.18)	47.67 (12.65)	52.22 (11.41)	47.88 (11.51)	48.38 (12.9)	48.33 (12.59)
triglycerides (mg/dl)*	166.28 (111.4)	152.08 (106.87)	187.2 (123)	159.24 (74.38)	162.92 (78.25)	128.16 (71.03)	185.18 (82.73)	166.54 (115.08)	164.39 (79.49)
CRP (mg/l)*	3.53 (5.96)	3.31 (5.81)	3.9 (6.17)	3.14 (7.21)	3.52 (5.5)	2.47 (7.66)	3.62 (4.71)	3.56 (5.97)	3.39 (5.87)
HbA1c (%)*	6.41 (1.25)	6.39 (1.2)	6.36 (1.3)	6.78 (1.44)	6.88 (1.2)	5.9 (0.59)	7.02 (1.38)	6.38 (1.24)	6.83 (1.27)

* mean (sd)

Table S2. Summary of immune response phenotypes with active bococizumab treatment for genetic analysis

	all studies		SPIRE-CVO*		SPIRE lipids*	
Clinical variable (units)	N	N postive (%) or median (IQR)	N	N postive (%) or median (IQR)	N	N postive (%) or median (IQR)
median (IQR) f/u (months)		343 (218-412)		322 (192-422)		406 (401-407)
ADA positive	8774	2841 (32.4%)	7725	2331 (30.2%)	1049	510 (48.6%)
ADA max titer^	2841	8.7 (7.5-10.3)	2331	8.7 (7.5-10.3)	510	8.9 (7.6-10.5)
ADA titer top 10% [N (%)]	2841	286 (10.1%)	2331	221 (9.5%)	510	65 (12.7%)
NAb positive	1048	312 (29.8%)	NA	NA	1048	312 (29.8%)
NAb max titer^	312	3.2 (2.8-5.0)	NA	NA	312	3.2 (2.8-5.0)

*SPIRE CVO = SPIRE1 + SPIRE 2, SPIRE lipids = SPIREHR + SPIRELDL + SPIREFH + SPIRELL.

^raw units, i.e. not $\log_2$

Table S3. Summary of immune response phenotypes across SPIRE substudies

	SPIRE sub-sample					
Clinical variable (units)	SPIRE1	SPIRE2	SPIREHR	SPIRELDL	SPIREFH	SPIRELL
median (IQR) f/u (months)^	261(140-386)	364(295-462)	406(401-407)	406(404-407)	407(406-414)	405(399-406)
ADA positive (% total)	25.90%	36.30%	48.80%	46.70%	52%	51.20%
ADA max titer^	8.5 (7.4-10)	8.8 (7.5-10.4)	8.6 (7.6-10.1)	8.9 (7.5-10.7)	8.1 (7.3-10.2)	9.1 (7.8-10.7)
ADA titer top 10% (%)	8.60%	10.40%	10.80%	14.30%	7.50%	13.20%
NAb posneg positive (%)	NA	NA	27.60%	27.90%	29.40%	35.30%
NAb max titer^	NA	NA	3.7 (2.8-5.4)	3.2 (2.8-4.8)	3.2 (2.3-4.3)	3.2 (2.8-5.1)

^median (IQR), N; *SPIRE CVO = SPIRE1 + SPIRE 2, SPIRE lipids = SPIREHR + SPIRELDL + SPIREFH + SPIRELL

^raw units, i.e. not $\log_2$

Table S4. Association* of clinical covariates with immune response phenotypes

Covariate	ADA positive (N=8774)	ADA max titer (N=2841)	ADA top 10% (N=2841)	NAb max titer (N=312)	NAb positive (N=1041)
Baseline age (years)	-0.022 (0.003), 3.93e-19	-0.001 (0.002), 6.03e-01	-0.007 (0.007), 2.70e-01	-0.002 (0.006), 7.81e-01	-0.009 (0.007), 2.03e-01
Sex (0=male/1=female)	0.065 (0.011), 2.83e-09	0.198 (0.04), 5.81e-07	0.027 (0.012), 2.63e-02	0.025 (0.115), 8.26e-01	0.093 (0.029), 1.61e-03
BMI (kg/m^2)	0.008 (0.004), 4.53e-02	0.007 (0.003), 4.64e-02	0.031 (0.011), 4.30e-03	0.013 (0.01), 2.14e-01	0.021 (0.012), 7.39e-02
current smoking (no=0/yes=1)	0.044 (0.012), 1.91e-04	0.078 (0.043), 7.02e-02	0.016 (0.013), 2.37e-01	-0.126 (0.155), 4.16e-01	-0.023 (0.038), 5.38e-01
diabetes (no=0/yes=1)	-0.014 (0.01), 1.65e-01	0.02 (0.038), 5.99e-01	0.024 (0.011), 3.98e-02	-0.109 (0.117), 3.54e-01	0.003 (0.029), 9.05e-01
LDL-C (mg/dl, continuous)	0.001 (0.001), 2.43e-01	0.001 (0.001), 3.46e-02	0.002 (0.002), 1.99e-01	0 (0.002), 9.93e-01	0.002 (0.002), 3.42e-01
HDL-C (md/dl, continuous)	0.002 (0.002), 3.52e-01	0.001 (0.001), 3.28e-01	-0.004 (0.005), 4.66e-01	-0.001 (0.004), 7.44e-01	0.007 (0.005), 1.77e-01
triglycerides (md/dl, log transformed, continuous)	0.001 (2e-04), 9.70e-04	7e-05 (2e-04), 7.04e-01	0.001 (5e-04), 4.59e-02	3e-04 (0.001), 7.42e-01	3e-04 (0.001), 7.55e-01
CRP (log transformed, continuous)	-0.007 (0.004), 1.13e-01	0.005 (0.004), 1.39e-01	0.015 (0.01), 1.18e-01	0.002 (0.01), 8.06e-01	0.007 (0.011), 4.99e-01
HbA1c (%)	-0.032 (0.02), 1.17e-01	0.032 (0.017), 5.35e-02	0.109 (0.05), 2.92e-02	0.111 (0.062), 7.67e-02	-0.057 (0.071), 4.24e-01

*beta (SE), p-value from logistic (ADA/NAb positive or ADA top 10%) or linear (ADA/NAb max titer) including indicateor variable for study

Table S5. Effects and significance* of MHC index variants across phenotypes

	index variant					
phenotype	AA_DRB1_120_32657518_N	AA_DQB1_75_32740612	rs3093664	rs7756741	rs3763313	AA_DQB1_71_32740624_KD
ADA_POSNEG_SUBJ	0.451 (1.7e-17)	0.219 (4.8e-09)	0.339 (2.7e-08)	0.189 (5.8e-08)	0.012 (7.5e-01)	0.219 (4.8e-09)
ADA_TITER_MAX	0.138 (2.0e-03)	0.200 (7.2e-12)	0.080 (8.4e-02)	0.194 (1.7e-12)	0.169 (4.0e-08)	0.200 (7.2e-12)
HIGH.ADA.TOPTEN	0.366 (2.9e-02)	0.541 (5.2e-09)	-0.008 (9.6e-01)	0.385 (2.4e-05)	0.444 (5.6e-06)	0.541 (5.2e-09)
NAB_POSNEG_SUBJ	0.433 (6.2e-03)	0.474 (1.1e-05)	0.142 (4.3e-01)	0.414 (5.5e-05)	0.258 (2.1e-02)	0.474 (1.1e-05)
NAB_TITER_MAX	0.139 (3.3e-01)	0.278 (9.5e-04)	0.094 (5.1e-01)	0.284 (7.2e-04)	0.116 (2.2e-01)	0.278 (9.5e-04)

*As beta coefficient (p-value)

**Table S6. Interaction with sex for top associations**			
phenotype	variant	main effect beta (SE), p	sex interaction-p
ADA pos/neg	AA_DRB1_120_32657518_N	-0.09(0.01), 1.69E-13	0.79
	AA_DQB1_75_32740612	0.04(0.01), 7.73E-06	0.61
	rs3093664	0.07(0.02),6.34E-06	0.86
ADA titer max	rs7756741	0.04(0.01), 1.02E-05	0.98
	rs3763313	0.01(0.01), 0.53	0.92
ADA titer top 10%	AA_DQB1_71_32740624_KD	0.04(0.01), 7.73E-06	0.61

^coded as 0=male, 1=female. Models also included age, smoking status and study

Table S7. Tests of non-additive associations at index variants in the MHC

			beta (SE), p		
phenotype	SNP	chr:pos	additive term*	dominance term^	p (2df)[@]
ADA posneg	AA_DRB1_120_32657518_N	6:32549539	0.50 (0.39-0.64), 1.75E-08	1.35 (1.04-1.75), 0.024	2.22E-16
	AA_DQB1_75_32740612	6:32632633	1.22 (1.11-1.33), 2.76E-05	1.06 (0.94-1.19), 0.38	2.61E-08
	rs3093664	6:31544641	1.25 (0.92-1.69), 0.15	1.14 (0.83-1.58), 0.42	1.71E-07
ADA max titer	rs7756741	6:32583197	0.19 (0.03), 2.84E-10	0.02 (0.04),0.60	2.84E-10
	rs3763313	6:32376470	0.17(0.04),1.61E-05	-0.01 (0.05),0.91	2.49E-07
ADA titer top 10%	AA_DQB1_71_32740624_KD	6:32632633	1.75 (1.44-2.12), 1.46E-08	0.93 (0.72-1.23),0.65	2.55E-08

*coded as 0=homozygote major allele, 1=heterozygote, 2=homozygote of minor allele; ^coded as 1=heterozygote, 0=otherwise. Models also included age, sex, eigenvectors and smoking status; [@]p-value for 2df genotype model

Table S8. Missense and nonsense proxies (r^2=0.9) for index variants			
index variant	proxy variant	chr:pos	A1/A2*
AA_DRB1_120_32657518_N		6:32549539	A/P
	AA_DRB1_96_32657590_Ex	6:32549611	P/A
	AA_DRB1_96_32657590_Q	6:32549611	P/A
	AA_DRB1_96_32657590_x	6:32549611	A/P
	AA_DRB1_96_32657590_Yx	6:32549611	P/A
	AA_DRB1_98_32657584_x	6:32549605	P/A
	AA_DRB1_104_32657566_x	6:32549587	P/A
	AA_DRB1_112_32657542_H	6:32549563	A/P
	AA_DRB1_112_32657542_Y	6:32549563	P/A
	AA_DRB1_120_32657518_x	6:32549539	P/A
	AA_DRB1_133_32657479_x	6:32549500	P/A
	AA_DRB1_140_32657458_x	6:32549479	P/A
	AA_DRB1_142_32657452_x	6:32549473	P/A
	AA_DRB1_149_32657431_x	6:32549452	P/A
	AA_DRB1_166_32657380_Q	6:32549401	P/A
	AA_DRB1_166_32657380_x	6:32549401	A/P
	AA_DRB1_180_32657338_L	6:32549359	A/P
	AA_DRB1_180_32657338_x	6:32549359	P/A
	AA_DRB1_181_32657335_x	6:32549356	P/A
AA_DQB1_75_32740612		6:32632633	L/V
	AA_DQB1_66_32740639	6:32632660	E/D
	AA_DQB1_67_32740636	6:32632657	V/I
	AA_DQB1_71_32740624_KT	6:32632645	A/P
	AA_DQB1_71_32740624_KD	6:32632645	P/A
	AA_DQB1_74_32740615_E	6:32632636	P/A
	AA_DQB1_75_32740612	6:32632633	L/V
rs7756741		6:32583197	A/T
	AA_DRB1_9_32660121_E	6:32552142	A/P
	AA_DRB1_11_32660115_SPL	6:32552136	A/P
	AA_DRB1_11_32660115_SV	6:32552136	P/A
	AA_DRB1_11_32660115_SVG	6:32552136	A/P
	AA_DRB1_11_32660115_SVL	6:32552136	A/P
	AA_DRB1_13_32660109_SY	6:32552130	P/A
	AA_DRB1_13_32660109_YF	6:32552130	A/P

*A1/A2=minor allele/major allele, A/P (or P/A) refers to absence (A) or presence (P) of amino acid(s) indicated by the last group of letters (which specify amino acids using the 1-letter code) in the variant designation. "x" refers to deletion of a residue. Other amino acid A1/A2 designations use the 1-letter code.

Table S9. Pairwise LD (D' and r^2)* for all index and conditional variants

				Variant																			
variant^	hg19 position	phenotypes[@]	nearest gene	rs29220	rs2267635	rs2240804	rs4713420	AA_C_9_31347600_S	rs2523453	rs3130062	rs3093664	rs9469069	rs3763313	rs9268500	rs9268543	rs2395171	rs17496549	AA_DRB1_120_32657518_N	AA_DRB1_13_32660109_FG	rs7756741	AA_DQB1_71_32740624_KD	rs3763355	rs3130215
rs29220	29589665	ADA postivie (8)	*GABBR1*	-	0.11	0.00	0.00	0.00	0.03	0.00	0.01	0.01	0.00	0.00	0.00	0.00	0.01	0.01	0.00	0.00	0.00	0.00	0.00
rs2267635	29592430	ADA postivie (9)	*GABBR1*	1.00	-	0.03	0.00	0.01	0.00	0.01	0.02	0.00	0.00	0.00	0.00	0.00	0.00	0.00	0.00	0.00	0.00	0.00	0.00
rs2240804	30920889	ADA titer max (5)	*HCG21*	0.04	0.45	-	0.01	0.02	0.00	0.09	0.07	0.00	0.00	0.00	0.01	0.00	0.00	0.00	0.00	0.00	0.01	0.00	0.01
rs4713420	30993566	ADA positive (10)	*MUC22*	0.15	0.09	0.32	-	0.00	0.00	0.00	0.00	0.00	0.00	0.00	0.00	0.00	0.00	0.00	0.00	0.00	0.00	0.00	0.00
AA_C_9_31347600_S	31239620	ADA titer max (4)	*HLA-C*	0.00	0.59	0.32	0.35	-	0.00	0.05	0.00	0.00	0.04	0.02	0.06	0.00	0.00	0.00	0.00	0.02	0.03	0.00	0.00
rs2523453	31368124	ADA titer max (2)	*MICA-AS1*	0.18	0.13	0.12	0.33	0.08	-	0.00	0.03	0.03	0.01	0.01	0.01	0.00	0.02	0.03	0.02	0.01	0.00	0.00	0.00
rs3130062	31525911	ADA positive (7)	*NFKBIL1*	0.07	0.32	0.38	0.08	0.39	0.07	-	0.10	0.01	0.00	0.01	0.02	0.00	0.00	0.00	0.00	0.02	0.04	0.00	0.00
rs3093664	31544641	ADA postivie (1)	*TNF*	0.21	0.17	0.64	1.00	0.04	0.44	1.00	-	0.00	0.01	0.00	0.01	0.00	0.01	0.01	0.03	0.01	0.00	0.00	0.00
rs9469069	31866417	ADA positive (5)	*C2*	0.32	0.04	0.43	0.00	0.52	0.67	0.73	1.00	-	0.01	0.00	0.00	0.00	0.00	0.00	0.04	0.02	0.03	0.00	0.00
rs3763313	32376470	ADA positive (6)	*BTNL2*	0.02	0.06	0.16	0.03	0.23	0.26	0.13	0.62	0.70	-	0.13	0.48	0.06	0.04	0.04	0.00	0.08	0.13	0.04	0.00
rs9268500	32376516	ADA positive (3)	*BTNL2*	0.09	0.22	0.31	0.62	0.46	0.52	0.53	0.40	0.74	1.00	-	0.27	0.00	0.01	0.01	0.00	0.08	0.03	0.09	0.00
rs9268543	32384800	ADA positive (4)	*BTNL2*	0.06	0.42	0.32	0.32	0.41	0.39	0.43	0.71	0.84	0.99	0.99	-	0.01	0.02	0.02	0.01	0.30	0.32	0.07	0.00
rs2395171	32394536	ADA titer max (7)	*HLA-DRA*	0.06	0.04	0.02	0.01	0.16	0.24	0.08	0.34	0.78	0.72	1.00	1.00	-	0.00	0.01	0.01	0.02	0.01	0.00	0.00
rs17496549	32409707	ADA titer max (3)	*HLA-DRA*	0.20	0.19	0.03	0.07	0.06	0.26	0.01	0.83	0.61	0.95	1.00	0.98	1.00	-	0.68	0.02	0.03	0.04	0.01	0.00
**AA_DRB1_120_32657518_N**	**32549539**	**ADA postive**	***HLA-DRB1***	0.15	0.52	0.05	0.05	0.06	0.34	0.13	0.79	0.77	0.92	1.00	0.95	1.00	0.89	-	0.04	0.07	0.05	0.01	0.00
AA_DRB1_13_32660109_FG	32552130	ADA titer max (1)	*HLA-DRB1*	0.08	0.12	0.02	0.01	0.01	0.20	0.04	0.32	0.59	0.20	0.15	0.44	1.00	0.66	1.00	-	0.30	0.05	0.00	0.00
**rs7756741**	**32583197**	**ADA titer max**	***HLA-DQB1***	0.04	0.04	0.09	0.21	0.15	0.09	0.22	0.29	0.59	0.35	0.92	0.96	0.97	0.73	0.99	0.70	-	0.60	0.05	0.00
**AA_DQB1_71_32740624_KD**	**32632645**	**ADA top 10%, nAb positive**	***HLA-DQB1***	0.02	0.26	0.23	0.02	0.19	0.02	0.39	0.32	0.52	0.36	0.48	0.80	0.98	1.00	1.00	0.24	0.95	-	0.07	0.00
rs3763355	32786881	ADA titer max (6)	*HLA-DOB*	0.06	0.11	0.10	0.00	0.19	0.11	0.15	0.15	0.30	0.45	0.35	0.43	0.04	0.89	0.82	0.43	0.66	0.62	-	0.00
rs3130215	33074962	ADA postive (2)	*HLA-DPB2*	0.06	0.01	0.10	0.01	0.00	0.04	0.02	0.06	0.32	0.07	0.10	0.15	0.10	0.11	0.08	0.12	0.01	0.00	0.12	-

*D' below and r^2 above diagonal. ^Index variants in bold font, conditional variants in plain font. [@]number in parenthesis refers to round of conditioning.

AA_DQB1_71_32740624_KD is a perfect proxy for AA_DQB1_75_32740612

Table S10. Associations with availale candidate MHC alleles

locus	candidate allele	agent	beta (SE), p^ ADA posneg	beta (SE), p^ NAb posneg
DRB1	03	TNFα	0.819 [0.693-0.968], 0.019	0.97 [0.60-1.57], 0.90
DRB1	03:01	γ-interferon/ TNFα	0.819 [0.693-0.968], 0.019	0.97 [0.60-1.57], 0.90
DQA1	05	TNFα	1.08 [0.97-1.19], 0.15	0.87 [0.64-1.18], 0.37
	0501		1.08 [0.98-1.20], 0.13	0.87 [0.64-1.18], 0.37

^Statistics from logistic model including age, sex, XX, and XX population eigenvectors

Table S11. Effects* of index SNP from conditional analysis on NAb and ADA status over follow-up for all available data

A. SNP association with ADA status over follow-up

		week:	4	12	24	36	48	52	58
		N:	1015	986	974	965	923	921	899
primary phenotype	variant	fr pos:	0.056	0.19	0.36	0.34	0.28	0.28	0.38
ADA top 10%	AA_DQB1_71_32740624_KD_P		1.64 (1.07-2.51), 2.2e-02	1.60 (1.23-2.08), 4.4e-04	1.34 (1.07-1.67), 9.6e-03	1.42 (1.13-1.78), 2.3e-03	1.59 (1.24-2.02), 2.1e-04	1.60 (1.25-2.03), 1.6e-04	1.25 (0.99-1.57), 5.9e-02
ADA posneg	AA_DQB1_75_32740612		1.64 (1.07-2.51), 2.2e-02	1.60 (1.23-2.08), 4.4e-04	1.34 (1.07-1.67), 9.6e-03	1.42 (1.13-1.78), 2.3e-03	1.59 (1.24-2.02), 2.1e-04	1.60 (1.25-2.03), 1.6e-04	1.25 (0.99-1.57), 5.9e-02
ADA posneg	AA_DRB1_120_32657518_N_A		0.62 (0.32-1.21), 1.6e-01	0.58 (0.39-0.87), 8.7e-03	0.82 (0.61-1.10), 1.8e-01	0.75 (0.55-1.01), 6.1e-02	0.69 (0.49-0.98), 3.6e-02	0.58 (0.40-0.83), 2.6e-03	0.71 (0.52-0.97), 3.2e-02
ADA posneg	rs3093664_G		0.94 (0.45-1.98), 8.8e-01	1.31 (0.86-2.00), 2.1e-01	1.40 (0.97-2.01), 7.3e-02	1.19 (0.82-1.72), 3.6e-01	1.05 (0.70-1.57), 8.2e-01	0.91 (0.60-1.37), 6.5e-01	1.22 (0.83-1.78), 3.1e-01
ADA titer max	rs3763313_A		1.62 (1.04-2.52), 3.4e-02	1.35 (1.04-1.76), 2.6e-02	1.00 (0.80-1.26), 9.8e-01	1.17 (0.93-1.47), 1.7e-01	1.14 (0.89-1.46), 2.9e-01	1.13 (0.88-1.44), 3.4e-01	1.02 (0.81-1.28), 8.7e-01
ADA titer max	rs7756741_A		1.52 (0.99-2.34), 5.3e-02	1.60 (1.24-2.07), 3.3e-04	1.24 (1.00-1.54), 4.7e-02	1.28 (1.03-1.59), 2.5e-02	1.41 (1.11-1.79), 4.2e-03	1.33 (1.05-1.68), 1.6e-02	1.21 (0.97-1.50), 9.5e-02

B. SNP association with NAb status over follow-up

		week:	4	12	24	36	48	52	58
		N:	1015	986	974	965	923	921	899
primary phenotype	variant	fr pos:	0.036	0.14	0.21	0.17	0.12	0.12	0.14
ADA top 10%	AA_DQB1_71_32740624_KD_P		1.78 (1.05-3.00), 3.1e-02	1.63 (1.21-2.19), 1.3e-03	1.53 (1.18-1.97), 1.2e-03	1.88 (1.43-2.48), 6.7e-06	2.07 (1.49-2.87), 1.4e-05	1.90 (1.39-2.61), 6.6e-05	1.72 (1.26-2.35), 5.7e-04
ADA posneg	AA_DQB1_75_32740612_L		1.78 (1.05-3.00), 3.1e-02	1.63 (1.21-2.19), 1.3e-03	1.53 (1.18-1.97), 1.2e-03	1.88 (1.43-2.48), 6.7e-06	2.07 (1.49-2.87), 1.4e-05	1.90 (1.39-2.61), 6.6e-05	1.72 (1.26-2.35), 5.7e-04
ADA posneg	AA_DRB1_120_32657518_N_A		0.68 (0.31-1.52), 3.5e-01	0.53 (0.32-0.86), 9.7e-03	0.71 (0.49-1.02), 6.7e-02	0.67 (0.45-1.02), 5.9e-02	0.62 (0.37-1.04), 6.9e-02	0.56 (0.34-0.93), 2.6e-02	0.69 (0.43-1.09), 1.1e-01
ADA posneg	rs3093664_G		0.48 (0.14-1.62), 2.4e-01	1.37 (0.86-2.19), 1.9e-01	0.99 (0.64-1.51), 9.5e-01	1.07 (0.68-1.68), 7.8e-01	1.19 (0.70-2.00), 5.2e-01	1.08 (0.64-1.82), 7.8e-01	1.34 (0.82-2.18), 2.4e-01
ADA titer max	rs3763313_A		1.87 (1.09-3.19), 2.3e-02	1.35 (1.00-1.83), 4.9e-02	1.28 (0.98-1.66), 6.5e-02	1.29 (0.98-1.71), 7.4e-02	1.28 (0.92-1.80), 1.5e-01	1.17 (0.84-1.63), 3.5e-01	1.28 (0.93-1.77), 1.3e-01
ADA titer max	rs7756741_A		1.66 (0.98-2.82), 6e-02	1.76 (1.31-2.36), 1.6e-04	1.45 (1.13-1.86), 3.9e-03	1.64 (1.25-2.16), 3.5e-04	1.79 (1.29-2.48), 4.6e-04	1.74 (1.27-2.37), 5.5e-04	1.64 (1.21-2.23), 1.4e-03

*beta (SE) p-value from linear (ADA/NAb max titer) or logistic (ADA/NAb positive) regression models with encoding of variant minor allele and covariates age, sex, smoking status, substudy
